# Characterization of the FLAVIN-BINDING, KELCH REPEAT, F-BOX 1 Homolog *SlFKF1* in Tomato as a Model for Plants with Fleshy Fruit

**DOI:** 10.3390/ijms22041735

**Published:** 2021-02-09

**Authors:** Tomoki Shibuya, Manabu Nishiyama, Kazuhisa Kato, Yoshinori Kanayama

**Affiliations:** 1 Faculty of Life and Environmental Science, Shimane University, Matsue 690-8504, Japan; tomoki.s.t.f@gmail.com; 2 Graduate School of Agricultural Science, Tohoku University, Aoba-ku, Sendai 980-8572, Japan; manabu.nishiyama.c3@tohoku.ac.jp

**Keywords:** *Solanum lycopersicum*, Solanaceae, FLAVIN-BINDING, KELCH REPEAT, F-BOX 1, blue light, flowering, ripening, lycopene

## Abstract

FLAVIN-BINDING, KELCH REPEAT, F-BOX 1 (FKF1) is a blue-light receptor whose function is related to flowering promotion under long-day conditions in *Arabidopsis thaliana*. However, information about the physiological role of FKF1 in day-neutral plants and even the physiological role other than photoperiodic flowering is lacking. Thus, the FKF1 homolog SlFKF1 was investigated in tomato, a day-neutral plant and a useful model for plants with fleshy fruit. It was confirmed that SlFKF1 belongs to the FKF1 group by phylogenetic tree analysis. The high sequence identity with *A. thaliana* FKF1, the conserved amino acids essential for function, and the similarity in the diurnal change in expression suggested that SlFKF1 may have similar functions to *A. thaliana* FKF1. CONSTANS (CO) is a transcription factor regulated by FKF1 and is responsible for the transcription of genes downstream of *CO*. *cis*-Regulatory elements targeted by CO were found in the promoter region of *SINGLE FLOWER TRUSS (SFT)* and *RIN*, which are involved in the regulation of flowering and fruit ripening, respectively. The blue-light effects on *SlFKF1* expression, flowering, and fruit lycopene concentration have been observed in this study and previous studies. It was confirmed in RNA interference lines that the low expression of *SlFKF1* is associated with late flowering with increased leaflets and low lycopene concentrations. This study sheds light on the various physiological roles of FKF1 in plants.

## 1. Introduction

Cryptochrome (CRY) and phototropin are blue-light receptors in plants [1]. Studies using *Arabidopsis thaliana* have shown that these photoreceptors control plant responses, including de-etiolation, hypocotyl elongation, and photoperiodic flowering by CRY [2,3,4] and phototropism, stomatal opening, and chloroplast localization by phototropin [5,6,7,8], indicating that their functions are diverse. Even in long-day plants other than *A. thaliana*, flowering promotion by blue light, which is supposed to involve CRY, has been reported in *Petunia* and *Eustoma* [9,10].

There are other blue-light receptors, such as FLAVIN-BINDING, KELCH REPEAT, F-BOX 1 (FKF1), and ZEITLUPE (ZTL), whose function is reportedly related to flowering promotion under long-day conditions in *A. thaliana*. FKF1 and ZTL proteins control the function of CONSTANS (CO) antagonistically [11], and here, we focus on FKF1. FKF1 interacts with GIGANTEA (GI) in a blue-light-dependent manner in *A. thaliana* and induces the degradation of CYCLING DOF FACTOR 1 (CDF1), which suppresses *CO* transcription, and its family proteins [12,13,14,15]. FKF1 also stabilizes the CO protein by suppressing its degradation by CONSTITUTIVE PHOTOMORPHOGENIC1 (COP1) and SUPPRESSOR OF PHYA-105 (SPA) [16,17,18,19]. Furthermore, FKF1 promotes *FLOWERING LOCUS T* (*FT*) transcription by inducing the degradation of CDF1, which is the transcriptional repressor of *FT* [16]. In this way, FKF1 plays an essential role in flowering promotion by blue light. As FKF1 in long-day plants, it is suggested that the *Gypsophila* FKF1 homolog is involved in flowering promotion, besides *A. thaliana* [20,21]. Reportedly, OsFKF1 promotes flowering in rice, a short-day plant, regardless of the photoperiod condition [22]. The FKF1 homolog is also considered to play an important role in the developmental phase transition of liverwort [23]. In addition, FKF1 and ZTL regulate the clock period by ubiquitination [24].

Although FKF1 has been reported as a negative regulator of cellulose synthesis [25], there are still few reports on its physiological roles other than flowering promotion. *FKF1* in *A. thaliana* is expressed in the vascular bundle sheath of leaves in relation to flowering control. However, its expression is also found in other tissues, including cotyledons, leaves, guard cells, and root tips, and their physiological importance remains unknown [26,27]. Additionally, FKF1-like sequences are conserved on the genomes of many plant species regardless of photoperiod responsiveness; short-day plants rice and soybean have phylogenetically FKF1-orthologous genes [22,28]. A few reports have suggested the possible functions of FKF1, such as stem and root growth and potassium response [29,30,31]. From these findings, it is expected that FKF1 plays various roles in plants.

Tomato is generally considered as one of the day-neutral plants, whose FKF1 has not been investigated well, and can also be used as a useful model for plants with fleshy fruit, which has accumulated data for bioinformatics. Hence, in this study, the sequence and physiological roles of the tomato *FKF1* homolog *SlFKF1* were analyzed.

## 2. Results

### 2.1. SlFKF cDNA Sequence

**Figure 1:**
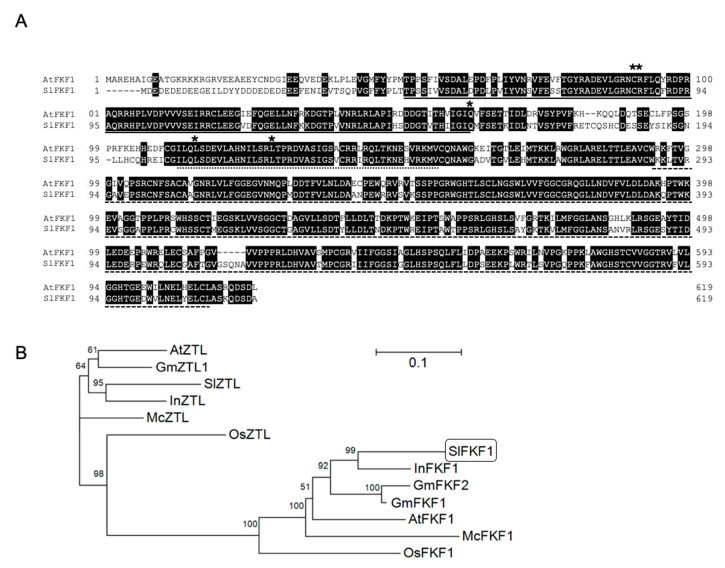
(**A**) Amino acid sequence alignment of SlFKF1 and *A. thaliana* FLAVIN-BINDING, KELCH REPEAT, F-BOX 1 (FKF1). Identical amino acids are shown in black boxes. Asterisks indicate the amino acids essential for the function of FKF1 [15,16,32]. (**B**) A phylogenetic tree based on the amino acid sequences of FKF1 and ZEITLUPE (ZTL) homologs from various species. LOV/PAS, F-box, and KELCH-repeat domain are shown on continuous, dotted, and broken lines, respectively, according to InterPro. The tree was constructed by the neighbor-joining method after sequence alignment using the Clustal W program. Branch numbers refer to the percentage of replicates that support the branch using the bootstrap method (1000 replicates). The scale bar corresponds to 0.1 amino acid substitutions per residue. Table S2 shows the accession numbers of the proteins used to construct the phylogenetic tree.

Using a BLAST search for a sequence orthologous to the amino acid sequence of *A. thaliana* FKF1, only one *FKF1*-like gene (XM_004228691.3) was found to be present in the tomato genome. This gene was labeled *SlFKF1*. The cDNA of *SlFKF1* was prepared from cv. Micro-Tom by reverse transcription–polymerase chain reaction (RT-PCR) and sequenced. Consequently, its sequence was the same as that on the database. On the alignment based on the amino acid sequence, SlFKF1 was 75.1% identical to *A. thaliana* FKF1, and the amino acids essential for the function of FKF1 were also conserved in SlFKF1 [15,16,32] (Figure 1A). As a result of a phylogenetic tree analysis based on alignment, including ZTL groups having the same domain structure as FKF1 but different functions, it was deduced that SlFKF1 belongs to the FKF1 group (Figure 1B).

### 2.2. Expression Analysis of SlFKF1

**Figure 2:**
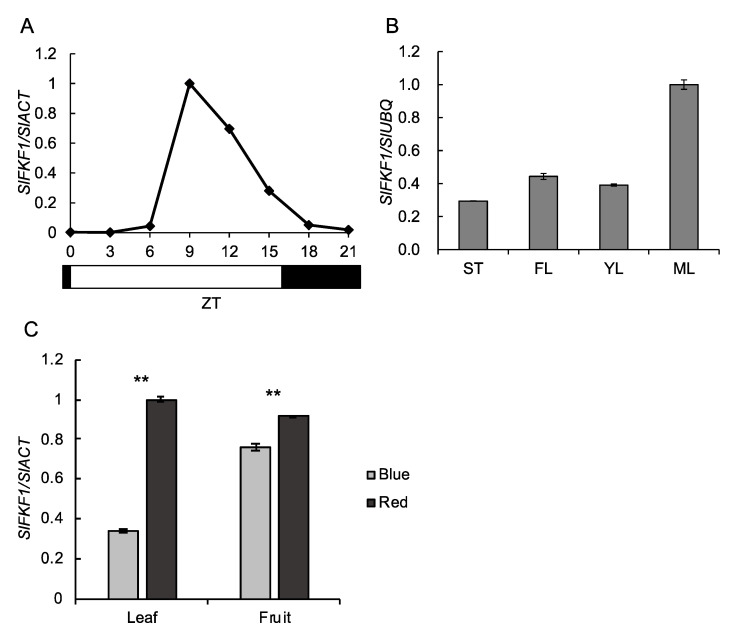
Diurnal change in the expression level of *SlFKF1* in leaves (**A**); expression level of *SlFKF1* in stems (ST), flowers (FL), young leaves (YL), and mature leaves (ML) (**B**); and expression level of *SlFKF1* in leaves and fruit under blue and red light (**C**). Total RNA was prepared from the wild-type plants of cv. Micro-Tom tomato. The relative expression levels were normalized against *SlUBQ* or *SlACT* with standard errors (*n* = 3), and the maximum level of the transcripts was set at 1.0. Values with ** are significantly different between blue and red light in each organ, according to Welch’s *t*-test (**C**).

*SlFKF1* expression in the wild-type cv. Micro-Tom tomato showed clear diurnal change under a 16 h day length (Figure 2A). The expression was very low from the dark period to the first 6 h of the light period and then increased. The expression peak of *SlFKF1* was shown at Zeitgeber time (ZT) 9. This expression pattern was similar to that of *A. thaliana FKF1* [13]. *SlFKF1* expression was observed in all organs tested in Figure 2B and was higher in mature leaves than in other organs. Furthermore, the effect of light quality on *SlFKF1* expression was investigated in leaves and fruit (Figure 2C). High expression levels were found in the fruit, similar to those in the leaves. The expression levels were lower under blue light in both organs.

### 2.3. Effect of Light Quality on Lycopene Concentration

**Figure 3:**
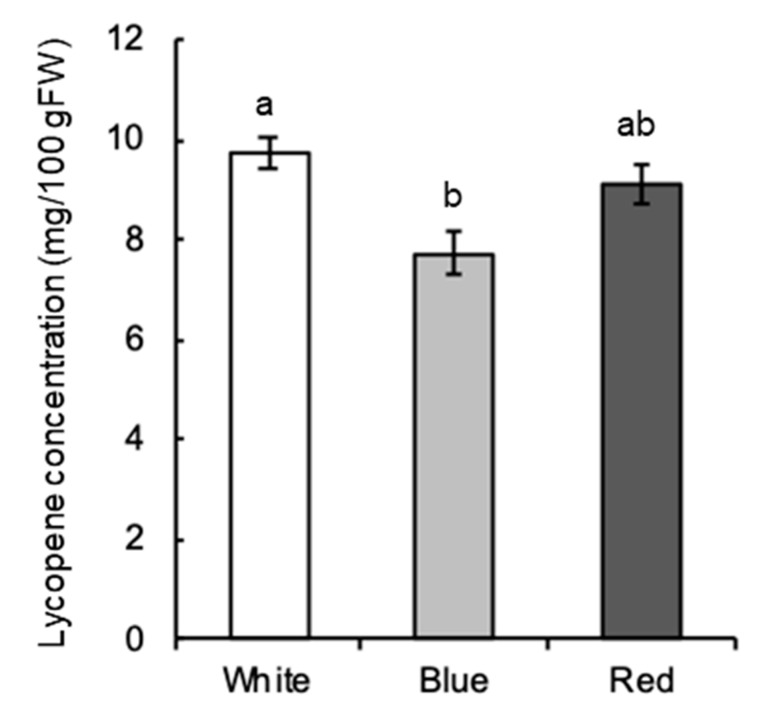
Lycopene concentration in fruit under white (W), blue (B), and red (R) light. Lycopene was extracted from the wild-type plants of cv. Micro-Tom tomato. Values indicate means with standard errors (*n* = 3). *p* < 0.01, values with different letters between treatments, according to Tukey’s test.

The effect of blue light on lycopene concentration, a major pigment in tomato fruit and a functional component [33], was investigated. Consequently, the lycopene concentration was lower under blue light than under white light, although it was not significantly different between white light and red light (Figure 3).

### 2.4. Analysis of SlFKF1 Promoter Sequences

Because FKF1 regulates downstream genes via the transcription factor CO [1,16], the possibility of regulating important factors related to flowering, ripening, and pigment synthesis was investigated. Putative CO-binding motifs were searched in the promoter regions of *FT*, *RIN*, and *PSY* tomato homologs, which are key factors for flowering promotion, fruit ripening control, and lycopene synthesis, respectively (Table 1). These genes were analyzed because *SINGLE FLOWER TRUSS* (*SFT*) is the tomato homolog of *FT* [34], and *RIN* and *PSY* are well-known important traits of tomato fruit development as factors governing the ripening process and carotenoid accumulation, respectively [35]. The motifs were found in *SFT*, *PSY1*, *PSY2,* and *RIN* promoters, whereas they were not found in the promoter of *PSY3* in the tomato genome.

### 2.5. Transformation Experiments

**Figure 4:**
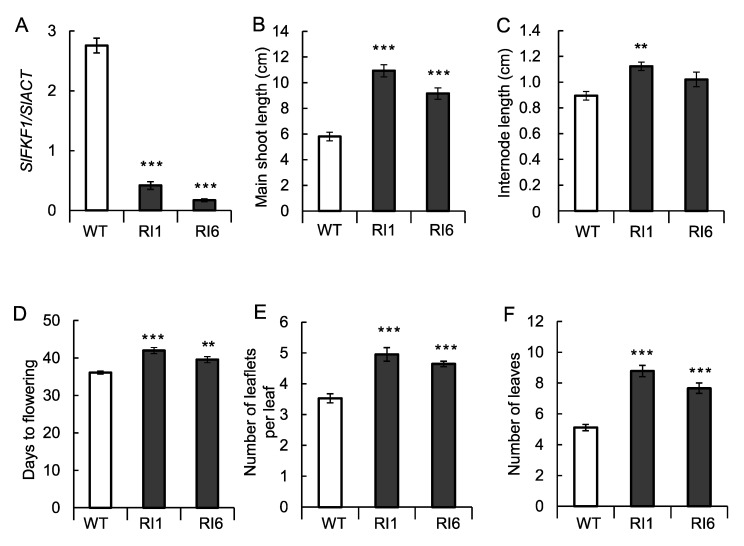
SlFKF1 mRNA levels and growth of SlFKF1 RNA interference (RNAi) lines (RI) and wild type (WT). SlFKF1 mRNA levels (**A**) were measured with the main shoot length (**B**) and internode length (**C**). Days to flowering indicate the number of days to first inflorescences from sowing (**D**). The number of leaflets per leaf was also measured (**E**). The number of leaves indicates the number of leaves to first flowers (**F**). Values indicate means with standard errors (*n* = 3 in A and *n* = 9 in **B**–**F**). *** *p* < 0.001 and ** *p* < 0.01, significantly different from WT, according to Dunnett’s test.

**Figure 5:**
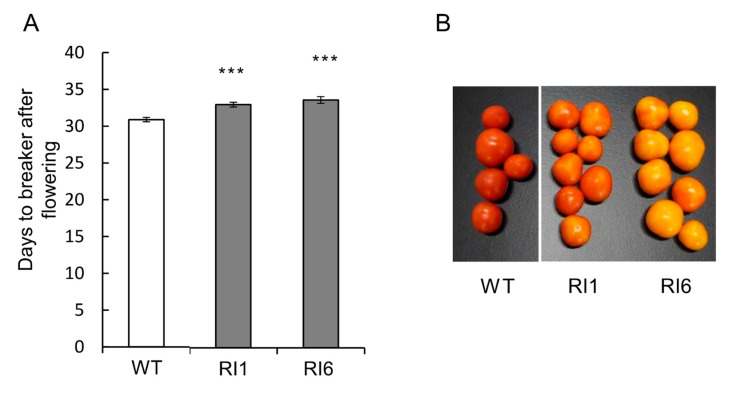
Number of days from flowering to the breaker stage (**A**) and fruit coloration 10 days after the breaker stage (**B**) in SlFKF1 RNAi lines (RI) and wild type (WT). Values indicate means with standard errors (*n* = 12–26). *** *p* < 0.01, significantly different from WT, according to Dunnett’s test.

SlFKF1 RNA interference (RNAi)-suppressed tomato plans were prepared to confirm the promoter analysis results above. The RNAi lines were differentiated from independent transformation events and produced normal seeds for phenotypic observations. The expression of each transgenic line at the expression peak of *SlFKF1* (ZT9) was investigated, and it was confirmed that expression-suppressed lines with RNAi have lower expression than the wild type (Figure 4A). The flowering in RNAi lines was investigated, and the number of days and leaves until flowering increased in RNAi lines compared to the wild type, suggesting that flowering was delayed by the suppression of *SlFKF1* expression (Figure 4D,F). Another interesting phenotype of RNAi lines was the increased number of leaflets (Figure 4E). The main stem length increased (Figure 4B), and this increase was not accompanied by an increase in internode length but the number of leaves (Figure 4C,F). In fact, because of the long main shoot length and a large number of leaves and leaflets, the RNAi line appeared to have a large plant volume (Figure S1). Regarding fruit coloration, the number of days from flowering to the breaker stage increased, and the degree of coloring was lower in RNAi lines than in the wild type 10 days after the beaker stage (Figure 5A,B).

## 3. Discussion

FKF1 has three domains: LOV, F-box, and KELCH repeat. In *A. thaliana*, it forms a gene family with ZTL [20]. ZTL has been reported to have a different function from FKF1 and is involved in the decomposition of TIMING OF CAB EXPRESSION 1 (TOC1), one of the components of the circadian clock [36,37,38]. The phylogenetic tree analysis results, including FKF1 and ZTL homologs, confirmed that SlFKF1 belongs to the FKF1 group. The high sequence identity with *A. thaliana* FKF1, the conserved amino acids essential for function, and the similarity in the diurnal change in expression suggested that SlFKF1 may have similar functions to *A. thaliana* FKF1. SlFKF1 was expressed not only in leaves, which are the photoreceptive organs for photoperiodic flowering control, but also in various organs, and its expression level was also high in fruit. Because the flowering of tomato is not affected by the photoperiod and the role of FKF1 in fruit has not been reported so far, in tomato, the physiological role of FKF1 other than in photoperiodic flowering should be examined.

CO is a transcription factor regulated by FKF1 and is responsible for the transcription of genes downstream of *CO* [1,16]. A CO-responsive element targeted by CO was reported by Tiwari et al. [39], and its CCACA core motif was identified by Gnesutta et al. [40]. SFT is the tomato homolog of FT involved in flowering promotion as a florigen [34], and RIN and PSY play important roles in governing the ripening process and carotenoid accumulation [35]. Therefore, it is possible that the FKF1-CO-mediated pathway regulates the expression of these genes and affects these developmental processes in tomato.

The heterozygotes of wild-type and mutant alleles of *SFT* in a determinant cultivar showed an approximately twofold increase in yield [41]. Thus, the physiological and agricultural importance of the *FT*-related flowering pathway in day-neutral plants is something of interest. A CO *cis*-element was found in the promoter region of *SFT*. Late flowering and increased leaflets were commonly observed in the *sft* mutant [42,43] and SlFKF1 RNAi lines, suggesting the relationship between *SlFKF1* and *SFT*. In *A. thaliana*, FKF1 is considered a blue-light receptor responsible for promoting flowering under long-day conditions, whereas in rice, a short-day plant, the FKF1 homolog promotes flowering regardless of day length [22]. The results suggested that an FKF1 homolog may also function in the flowering pathway of day-neutral plants. *A. thaliana* FKF1 promotes flowering by suppressing the function of the COP1/SPA system that degrades the CO protein [17,18,19], and tomato likely has a similar mechanism. In contrast, it has been proposed in *A. thaliana* that FKF1 positively regulates the gibberellin (GA) signal through the degradation of the DELLA protein and activates the GA-dependent flowering promotion pathway [44]. However, in tomato, because GA rather suppresses flowering [45], it would not be possible to apply this *A. thaliana* model to tomato.

Because SlFKF1 expression was suppressed by blue light, the effect of blue light on lycopene concentration was investigated for comparison. In this study, the effect of blue-light irradiation on lycopene concentration in fruit was measured during cultivation. Although the effects of blue-light irradiation on lycopene concentration have been investigated in previous reports [46,47], their experimental conditions were different from this study. The effects of supplemental blue light over a long period after flowering in a greenhouse [46] and blue-light irradiation after harvest [47] have been investigated in previous reports. In addition, the effects of blue light on lycopene concentration are different between these reports. In this study, to limit the effect on vegetative growth, after cultivation under white light, the effect was investigated by irradiating blue light from the breaker only for 10 days. As a result, this study and a previous report [47] showed low fruit lycopene concentrations under blue light. Collectively, it is likely that blue light alone for a relatively short period negatively affects lycopene accumulation in tomato fruit.

The fruit lycopene concentration was low under blue light, as described above, and there are *cis*-regulatory elements targeted by CO in the promoter regions of RIN and PSY. Additionally, fruit *SlFKF1* expression was low under blue light. Because there are reports that light quality regulates growth and environmental response through the regulation of photoreceptor gene expression [48,49], low *SlFKF1* expression might be related to low lycopene concentration under blue light as one possible mechanism. It was confirmed in RNAi lines that low expression of *SlFKF1* is associated with low concentrations of lycopene. So far, the effect of blue light on tomato fruit color has assumed the involvement of CRY [50,51,52], and there have been few reports on FKF1. A similar control mechanism is possible for flowering because blue light delays flowering in tomato [53,54], and leaf *SlFKF1* expression is also low under blue light. It is interesting to note that blue light promotes flowering in *A. thaliana* and some other long-day plants in contrast to tomato [10,21,55].

A previous report suggested a relationship between auxin and FKF1 in the adventitious rooting of longan [30]. Auxin plays an important role in the growth and ripening of tomato fruit [56]. Another report suggested that potassium levels, which are important in fruit growth and quality, affect *FKF1* expression in banana roots [31]. Therefore, the function of SlFKF1 may be related to auxin signaling and potassium nutrition in fruit. Because other photoreceptors such as CRY and PHY are considered important agronomic traits [57], the assumed role of FKF1 in fruit crops is promising based on this study.

FKF1 is present in the genomes of a wide variety of species, whereas so far, knowledge about its role has been limited. Its role in flowering in day-neutral plants and even the physiological role other than flowering is discussed in this study using tomato as a model plant, while preliminary transcriptome analysis has shown that blue light induces the expression of several transcriptional regulation- and signal transduction-related genes during tomato fruit ripening [58]. Starting with this study, the varying physiological roles of FKF1, which have been comparatively unknown compared to phytochrome and CRY, will be elucidated.

## 4. Materials and Methods

### 4.1. Plant Materials

Tomato cv. Micro-Tom wild type was cultivated for cloning and expression analysis in a growth chamber (LH240SP; Nihon Ika Co., Ltd., Osaka, Japan) at 25 °C with a white fluorescent lamp in a 16-h photoperiod. Photosynthetic photon flux density (PPFD) was 100 μmol m^−2^ s^−1^, and Sumisoil N150 (Sumika Agro-tech Co., Ltd., Osaka, Japan) was used as cultivation soil. The plants were supplemented with nutrient solution (Hyponex Japan) every week. The cultivation was conducted with reference to Tsunoda et al. [59].

### 4.2. Sequence Analysis of Tomato FKF1 Homolog SlFKF1 cDNA

A BLAST search was performed to search for a candidate protein of SlFKF1 and an open reading frame (ORF), encoding it based on the amino acid sequence of FKF1 (AT1G68050) of *A. thaliana* FKF1 in Tomato Genome CDS (ITAG release 2.40) of the Sol Genomics Network (SGN; https://solgenomics.net accessed on 8 February 2021) and NW_004194292.1 and XM_004228691.1 of the National Center for Biotechnology Information (NCBI; https://www.ncbi.nlm.nih.gov accessed on 8 February 2021). Using a primer set designed based on the candidate sequence obtained from the database, its ORF sequence was cloned by PCR with the cDNA obtained by reverse transcription of RNA extracted from cv. Micro-Tom tomato leaves and confirmed by sequencing.

### 4.3. Expression Analysis

For a diurnal change in expression, mature leaves that were fully developed and not senesced were randomly collected every 3 h from the start of the light period (ZT0) to ZT21 at approximately 30 days after germination. For the expression analysis in stems, flowers, immature leaves, and mature leaves, samples were collected on ZT8 approximately 50 days after germination. For the expression analysis in leaves under blue and red light, the plants at 2 months after sowing were cultivated for 1 day under a 16 h photoperiod with red or blue light emitting diodes (LEDs) (CCS Inc., Kyoto, Japan), and leaves were sampled for RNA extraction. For the expression analysis in fruit under blue and red light, the plants at the fruit breaker stage were cultivated for 10 days under a 16 h photoperiod with red or blue LEDs, and the pericarp was sampled for RNA extraction. PPFD was 100 μmol m^−2^ s^−1^, and the samples were collected on ZT10. Red and blue LEDs had peaks at 655 and 470 nm, respectively. RNA was prepared from these samples using the RNeasy Plant Mini Kit (Qiagen) for real-time PCR. The removal of genomic DNA and reverse transcription were performed using the Quantiscript Reverse Transcription Kit (Qiagen) and the ReverTra Ace qPCR RT Master Mix (Toyobo). Real-time PCR was performed using the QuantiTect SYBR Green PCR Kit (Qiagen) and the THUNDERBIRD SYBR qPCR Mix (Toyobo) according to Ikeda et al. [60]. *SlACT* and *SlUBQ* were used as reference genes [61,62], and Table S1 shows the nucleotide sequences for the primers.

### 4.4. Alignment, Phylogenetic Analysis, and Promoter Analysis

Using the amino acid sequences of AtFKF1 and AtZTL as queries, a BLAST search was performed for non-redundant protein sequences (nr) of each plant species in the NCBI to search for homologs. For tomato FKF1, a BLAST search was also performed on tomato genome protein sequences (ITAG release 2.40) on the SGN to obtain locus information about matching proteins, and the gene model was confirmed by referring to genomic detail. A phylogenetic tree was created using the neighbor-joining method for the alignment obtained by executing Clustal W2 on Genetyx version 10 for FKF1 and ZTL amino acid sequences. Bootstrap probabilities were calculated by 1000 trials.

For promoter analysis, each gene was searched by a keyword and BLAST on the SGN, and locus names were confirmed. Next, the locus was searched, and an upstream 3000-base sequence was obtained from the genomic sequence of genomic detail using the function of Get flanking sequences on SL2.50ch07. Additionally, the position of the start codon was confirmed from the cDNA sequence and protein sequence of the genomic detail, and the start codon upstream 1500-base sequence was determined. The CO-binding sequence CCACA was searched from the obtained 1500-base sequence by the Text Search function on Genetyx version 14.

### 4.5. Determination of Lycopene Concentration

The plants at the fruit breaker stage were cultivated for 10 days under a 16 h photoperiod with red LEDs, blue LEDs, or a white fluorescent lamp, and the pericarp was sampled for the determination of lycopene concentration. PPFD was 100 μmol m^−2^ s^−1^, and the samples were collected on ZT10. Red and blue LEDs (CCS Inc.) had peaks at 655 and 470 nm, respectively. According to Ito and Horie [63], lycopene was extracted using dimethyl ether/methanol (7:3), filtrated with DSMIC JP 13 (Advantec), and quantified by measuring the absorbance at 505 nm.

### 4.6. Transformation Experiment

RNAi was used for expression suppression. A partial fragment (341 bp) of *SlFKF1* was amplified by RT-PCR using the cDNA of cv. Micro-Tom as a template and introduced into pBI-RNAi-GW (Inplanta Innovations, Inc., Yokohama, Japan), a vector for preparing an RNAi construct with the CaMV *35S* promoter. Table S1 shows the nucleotide sequences for the primers used to prepare these vectors. The transformation was outsourced to Inplanta Innovations. The cultivation of transformed tomato plants and expression analysis were performed as described above.

## Figures and Tables

**Table 1:** CONSTANS (CO)-responsive elements on the promoters of *SFT*, *RIN*, and *PSY* homologs.

Gene	Locus ^1^	Putative CONSTANS Responsive Element ^2^	Strand	Position of 1st C from ATG
*SFT*	Solyc03g063100	TTT**CCACA**AAA	Top	−379
*PSY1*	Solyc03g031860	TTC**CCACA**CTG	Bottom	−554
	Solyc03g031860	AAA**TGTGG**TGT	Bottom	−269
	Solyc03g031860	GTC**TGTGG**TCT	Bottom	−186
*PSY2*	Solyc02g081330	TTG**TGTGG**TCA	Bottom	−274
*PSY3*	Solyc01g005940	not found		
*RIN*	Solyc05g012020	CTA**CCACA**AGG	Top	−1049
	Solyc05g012020	ATG**TGTGG**CTA	Bottom	−701

^1^ Locus number in the Sol Genomics Network (SGN). ^2^ Bold letters indicate the core motif.

## Supplementary Materials

Can be found at https://www.mdpi.com/1422-0067/22/4/1735/s1. Table S1: List of primer sequences, Table S2: Accession numbers of proteins used for the phylogenetic tree analysis, Figure S1: Wild-type (WT) and SlFKF1 RNAi line (RI6) plants with ripe fruit.
