# Supplementary material for: Characterization of the FLAVIN-BINDING, KELCH REPEAT, F-BOX 1 Homolog SlFKF1 in Tomato as a Model for Plants with Fleshy Fruit
Source: Int J Mol Sci. 2021 Feb 9;22(4):1735. doi: 10.3390/ijms22041735 (PMC7914597; doi:10.3390/ijms22041735)
Supplement: Supplementary file 1 [file ijms-22-01735-s001.pdf]

Table S1. List of primer sequences.

	Gene	Sequences of forward (1st line) and reverse (2nd line) primers
Real-time PCR	*SlFKF1*	5′-ATAGCACATGCGTGGTGGGAG-3′
		5′-TTGCTCGCTAAGCACAATTCATAC-3′
	SlACT	5′-CACTGTATGCCAGTGGTCGT-3′
		5′-ACGTCCCTGACAATTTCTCG-3
	SlUBI3	5′- CACCAAGCCAAAGAAGATCA-3′
		5′- TCAGCATTAGGGCACTCCTT-3′
Transformation	*SlFKF1*	5′-GGGGACAAGTTTGTACAAAAAAGCAGGCTCTTGCCAACAGCGCTAAC-3′
		5′-GGGGACCACTTTGTACAAGAAAGCTGGGTCTTGTTCCTCCAACCACG-3′

Table S2. Accession numbers of proteins used for the phylogenetic tree analysis.

Name	Accession number
AtZTL	AT5G57360
GmZTL1	NM_001248927
SlZTL	XM_004242857
InZTL	AB265784
McZTL	AY371290
OsZTL	XM_015787692
SlFKF1	XM_004228691
InFKF1	KJ605442
GmFKF2	XM_003530876
GmFKF1	NM_001248957
AtFKF1	AT1G68050
McFKF1	AAQ73528
OsFKF1	XM_015761570

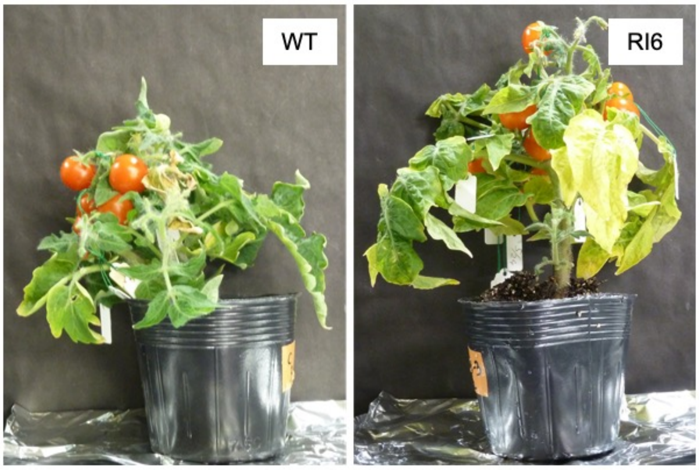

Figure S1. Wild-type (WT) and SlFKF1 RNAi line (RI6) plants with ripe fruit.
